# Application and Validation of Case-Finding Algorithms for Identifying Individuals with Human Immunodeficiency Virus from Administrative Data in British Columbia, Canada

**DOI:** 10.1371/journal.pone.0054416

**Published:** 2013-01-28

**Authors:** Bohdan Nosyk, Guillaume Colley, Benita Yip, Keith Chan, Katherine Heath, Viviane D. Lima, Mark Gilbert, Robert S. Hogg, P. Richard Harrigan, Julio S. G. Montaner, STOP HIV/AIDS Study Group

**Affiliations:** 1 BC Centre for Excellence in HIV/AIDS, Vancouver, British Columbia, Canada; 2 Division of AIDS, Faculty of Medicine, University of British Columbia, Vancouver, British Columbia, Canada; 3 Clinical Prevention Services, BC Centre for Disease Control, Vancouver, British Columbia, Canada; 4 School of Population and Public Health, University of British Columbia, Vancouver, British Columbia, Canada; 5 Faculty of Health Sciences, Simon Fraser University, Vancouver, British Columbia, Canada; IPO, Inst Port Oncology, Portugal

## Abstract

**Objective:**

To define a population-level cohort of individuals infected with the human immunodeficiency virus (HIV) in the province of British Columbia from available registries and administrative datasets using a validated case-finding algorithm.

**Methods:**

Individuals were identified for possible cohort inclusion from the BC Centre for Excellence in HIV/AIDS (CfE) drug treatment program (antiretroviral therapy) and laboratory testing datasets (plasma viral load (pVL) and CD4 diagnostic test results), the BC Centre for Disease Control (CDC) provincial HIV surveillance database (positive HIV tests), as well as databases held by the BC Ministry of Health (MoH); the Discharge Abstract Database (hospitalizations), the Medical Services Plan (physician billing) and PharmaNet databases (additional HIV-related medications). A validated case-finding algorithm was applied to distinguish true HIV cases from those likely to have been misclassified. The sensitivity of the algorithms was assessed as the proportion of confirmed cases (those with records in the CfE, CDC and MoH databases) positively identified by each algorithm. *A priori* hypotheses were generated and tested to verify excluded cases.

**Results:**

A total of 25,673 individuals were identified as having at least one HIV-related health record. Among 9,454 unconfirmed cases, the selected case-finding algorithm identified 849 individuals believed to be HIV-positive. The sensitivity of this algorithm among confirmed cases was 88%. Those excluded from the cohort were more likely to be female (44.4% vs. 22.5%; p<0.01), had a lower mortality rate (2.18 per 100 person years (100PY) vs. 3.14/100PY; p<0.01), and had lower median rates of health service utilization (days of medications dispensed: 9745/100PY vs. 10266/100PY; p<0.01; days of inpatient care: 29/100PY vs. 98/100PY; p<0.01; physician billings: 602/100PY vs. 2,056/100PY; p<0.01).

**Conclusions:**

The application of validated case-finding algorithms and subsequent hypothesis testing provided a strong framework for defining a population-level cohort of HIV infected people in BC using administrative databases.

## Introduction

The Public Health Agency of Canada (PHAC) estimated there were 11,700 individuals living with HIV/AIDS in British Columbia in 2011 [1], [2]. While medical care (HIV and non-HIV related), HIV testing, antiretroviral treatment and laboratory monitoring are fully subsidized by the provincial government for residents of BC [3], it has been suggested that as many as 26% of infected individuals are unaware of their sero-status. Furthermore, it has been estimated that as of 2008, as many as 40% of those who died of HIV-related causes did so without accessing treatment, and that as of 2010 only 42% of individuals eligible for HAART based on 2010 IAS-USA guidelines are actually receiving treatment [2], [4], [5].

As a result of continued incomplete access to care and the newly-discovered preventive benefits of Highly Active Antiretroviral Treatment (HAART) [6]–[8], the BC Ministry of Health (MoH), the BC Centre for Disease Control (CDC), the BC Centre for Excellence in HIV/AIDS (CfE), the BC Provincial Health Services Authority, Vancouver Coastal Health and the Northern Health Authority partnered in the Seek and Treat for Optimal Prevention of HIV and AIDS (STOP HIV & AIDS) pilot project in 2010, with a commitment to work collaboratively to increase HIV testing and address the gaps in the access to treatment and care within hard-to-reach populations with aim to reduce HIV-related morbidity, mortality, and transmission. A critical aspect of this initiative involves improved coordination and linkage of datasets capturing HIV testing, treatment and health resource utilization. The aims of this effort include assessing the economic implications of treatment scale-up and establishing a comprehensive monitoring system to assess rates of newly diagnosed cases access to care, and HAART uptake over time. The cascade of HIV care [9], [10] has become a focal point for implementation efforts to maximize the impact of HIV treatment at the individual and societal levels, highlighted by the World Health Organization as the central evaluation and monitoring metric for Treatment as Prevention in Global AIDS Response and Progress Reporting [11]. Identifying a complete cohort of known individuals living with HIV, and at each stage of the cascade of care over time is necessary to comprehensively evaluate these stated outcomes and thus fulfill the objectives of the project.

Health administrative data are defined as information collected for the purpose of health care management, often by government and health care providers [12], [13]. Because administrative data are not generated specifically for chronic disease surveillance or research purposes and there is no financial incentive associated with accuracy when physicians provide diagnostic data for billing, it is important to assess the validity of these data prior to deploying them for the aforementioned uses [14]. Isolated diagnostic codes associated with physician billing records have been shown to accurately identify patients with some chronic diseases [15], [16] but not others [17]–[20]. Since chronic diseases such as HIV/AIDS usually require multiple contacts with the health system to diagnose and treat, a single-visit diagnostic code is often insufficient to accurately identify cases [14]. Validation of algorithms used to identify patients within a given disease area or diagnosis is essential to avoid misclassification bias [21], which may threaten the internal validity and interpretation of study conclusions. Because of the risk of misclassification error associated with using administrative data for population-based research, the validation of these data has been identified as a priority by an international consortium of health services researchers [22]; in the context of HIV, the importance of monitoring HAART uptake and adherence to exploit the individual and public health benefits of treatment place an emphasis on comprehensive data collection mechanisms such as administrative databases. We sought to apply and validate a case-finding algorithm for identifying HIV cases using health administrative databases in British Columbia, Canada.

## Materials and Methods

### Study Population

The cohort of interest for analysis included all HIV positive persons aged 18 months of age or older who tested HIV positive or otherwise had some HIV-related record in at least one of the databases described in Table 1 between January 1st, 1995 and March 31st, 2010. Individuals were included if they were captured in the CDC HIV surveillance database (persons with a documented positive HIV test) or the CfE treatment registry (having at least one plasma viral load/CD4 test and/or receiving antiretroviral medications) or if they were identified within health administrative datasets held by the MoH (Medical Services Plan (MSP) database; Discharge Abstract Database (DAD)) as having received care for an HIV- or AIDS-related medical condition on at least one occasion. Additional linkages to provincial drug dispensation (BC PharmaNet database) and mortality records (BC Vital statistics database) were also available and employed in subsequent analyses to refine the cohort.

**Table 1:** Descriptions of databases used for cohort validation.

Database	Description
***BCCfE drug treatment program and laboratory disease registry***	The treatment program and clinical databases held at the BC-CfE include information on all individuals who have ever received antiretroviral treatment for HIV, including complete historical antiretroviral treatment records, HIV-related laboratory test records (80% of all CD4 tests provincially, all pVL, drug resistance tests), as well as information on demographics and mode of HIV transmission.
***BCCDC Provincial HIV/AIDS Surveillance Database***	The BC provincial HIV/AIDS surveillance database contains records of all individuals with a positive HIV test done in BC. It also captures information collected through an enhanced surveillance form for all persons with a newly diagnosed HIV infection.
***BC Ministry of Health (BCMoH) Administrative Databases***	
***Medical Services Plan (MSP) Database***	The MSP database includes records of all medical services provided by fee-for-service practitioners to individuals covered by British Columbia's Medical Insurance Plan (MSP) including laboratory and diagnostic procedures. It also includes encounter records for practitioners who are funded through areas such as Alternative Payment Branch (APP) or Primary care for the Population Based Funding (PBF) sites and claims records for the fee for service payments processed by MSP for the Insurance Corporation of British Columbia (ICBC) and Worksafe BC (WSBC). The dataset includes information on the dates, diagnoses, and types of outpatient care delivered throughout the study period, as well as the costs billed to the provincial Ministry of Health. Physician fee for service claims are reimbursed at the rates listed in the Medical Services Commission (MSC) Payment Schedule in accordance with the Schedule's Preamble rules.
***Discharge Abstract Database (DAD)***	Records for hospital discharges are included in the DAD file from the BC Ministry of Health. The DAD contains demographic, administrative and clinical information for acute, rehabilitation and day surgery patients in acute care hospitals in BC. The DAD does not include records for outpatient services such as emergency, clinic, diagnostic imaging and laboratory services.
**BC ** ***PharmaNet Database***	The BC PharmaNet database records all prescription drug dispensation in British Columbia. Data fields available included a de-identified patient ID, quantity dispensed (number of pills dispensed), de-identified prescriber code, cost of drugs dispensed, drug identification number, the date of the prescription, the length of the prescription (number of days supplied), drug dosage (quantity), de-identified prescriber code and the cost of drugs dispensed. Further information about the medication is also available including the generic code number (gcn) sequence number and American Hospital Formulary Service (AHFS) code (codes for grouping similar medications), name of the active ingredient, name of the product, dosage of the product and form of the medication (pill, capsule, etc.)
**BC Vital Statistics Database**	The BC Vital Statistics database includes fields on the date of death (year and month) as well as ICD-9 and ICD-10 codes identifying probable cause of death.

Individuals meeting the provincial HIV case definition, and testing HIV-positive for the first time in British Columbia, were included from the CDC database. This entails detection of HIV antibody by screening test (i.e., ELISA or Point of Care HIV test) followed by positive confirmatory test (i.e., Western Blot or Nucleic Acid Amplification Test), or Detection of HIV nucleic acid (RNA or DNA) or detection of p24 antigen with confirmation by neutralization assay, or isolation of HIV in culture. Tests were excluded when an individual chose non-nominal reporting as prescribed in the provincial *Communicable Disease Regulation*, where identifiers were insufficient for linkage. Individuals were identified in MoH datasets using ICD-9/10 diagnostic codes associated with HIV/AIDS (MSP: any ICD-9/10 code starting with ‘042’, ‘043’, ‘044’, ‘V08’, ICD-9 code 795.71 or ICD-9 codes starting with 795.8; DAD: all previous codes, in addition to ICD-10-CA codes B24, R75, Z21, B20–B23).

Database linkage was executed by data stewards in each collaborating agency and coordinated by the Vancouver Coastal Health Authority. Clients were matched to the client registry by provincial health number (PHN). PHNs are mandatory for all BC residents [23], and are not available to tourists or other non-residents. The final de-identified datasets were provided to the analysis team (CfE). A privacy impact assessment was completed for this study. Ethical approval was obtained through the UBC Behavioural Research Ethics Board (no. H08-02095).

### Procedures

#### Defining cohort exclusion criteria

Prior to application of the case-finding algorithm, we excluded individuals with one or more pVL tests with undetectable pVL and no other HIV-related records (hospitalization, physician claim, positive HIV test, AIDS-defining Illness or HAART). These cases typically represent instances where a pVL test was ordered for HIV diagnostic purposes among selected individuals whose antibody based test was HIV-negative. Those meeting the above criteria and also receiving antiretroviral medications through the BC PharmaNet database (rather than the CFE, from which HIV medications are universally covered) were considered to have received pre- or post-exposure prophylaxis and were also excluded. In addition, we excluded individuals receiving antiretrovirals (ARVs) for treatment of Hepatitis B, distinguished by the prescription of Lamivudine (3TC) or Tenofovir (TDF) or Truvada (TDF/FTC) alone as their only ARV ever prescribed (from the PharmaNet database), associated with no records of HAART treatment from the CFE. We also excluded infants receiving antiretrovirals up to 18 months of age, with no HIV-related records thereafter. Antiretroviral prophylaxis was prescribed up to a period of 18 months to prevent vertical HIV transmission [24]; cases with no HIV-related records after this point were considered to be successfully treated, and thus HIV-negative.

#### Application of case-finding algorithms

A previously-validated set of case-finding algorithms [14] were then applied to unconfirmed HIV positive cases. The algorithms defined decision rules for HIV classification based on varying quantities of HIV-related records in the DAD and MSP datasets; the entire study follow-up period was utilized in applying the algorithms.

Four case finding algorithms were considered: algorithm 1: indicating HIV-positivity with 3 HIV-related physician claims; algorithm 2: indicating HIV-positivity with 3 HIV-related physician claims OR 1 HIV-related hospital admission; algorithm 3: indicating HIV-positivity with 2 HIV-related physician claims or 1 HIV-related hospital admission; algorithm 4: indicating HIV-positivity with 1 HIV-related physician claim or 1 HIV-related hospitalization.

We defined confirmed HIV-positive cases as having records of either an HIV-positive test (CDC database) or records of pVL/CD4 tests or HIV-related medications in the CfE database; unconfirmed cases therefore only had HIV-related records in the DAD and MSP administrative datasets.

While we could not ascertain specificity (the proportion of HIV-negative individuals who were correctly identified as such), sensitivity (the proportion of HIV-positive individuals who were correctly identified as such) of each case-finding algorithm was assessed by applying the same case-finding algorithm to a subset of the cohort of individuals we classified as ‘gold standard’ HIV-positive cases, who had linked records from each of the data sources (CDC, CfE, MoH). These gold standard cases were used to assess the sensitivity of the algorithms.

#### Statistical Inference

The case finding algorithms could not provide an objective criterion for exclusion of unconfirmed cases. We therefore supported this analysis by testing a series of *a priori* hypotheses regarding the characteristics of cohorts considered for exclusion.

We specified five *a priori* hypotheses to assess the face validity of the algorithms. We hypothesized that proportion of females would be greater within a cohort of HIV-negative than HIV-positive individuals, as a result of high HIV prevalence among men who have sex with men in BC and elsewhere. Second, we hypothesized that the all-cause mortality rate would be lower within a cohort of HIV-negative compared to that of HIV-positive individuals. The mortality rate was defined as the number of deaths per 100 person years (100PY) of follow-up (estimated as the time between the first chronological health record to mortality or the end of follow-up). Finally, we tested three hypotheses regarding rates of health service utilization. Specifically, we hypothesized that the rate of outpatient care utilization (MSP claims), the rate of inpatient care, and the rate of pharmaceutical dispensations, all median individual rates per 100PY of follow-up (in this case estimated as the time between 1^st^ HIV-related diagnosis to death or censorship) would be lower in a cohort of HIV-negative compared to HIV-positive individuals. Statistical inference was conducted at an alpha level of 0.05, using non-parametric Kruskal-Wallis tests. All analyses were conducted using SAS version 9.2.

## Results

**Figure 1:**
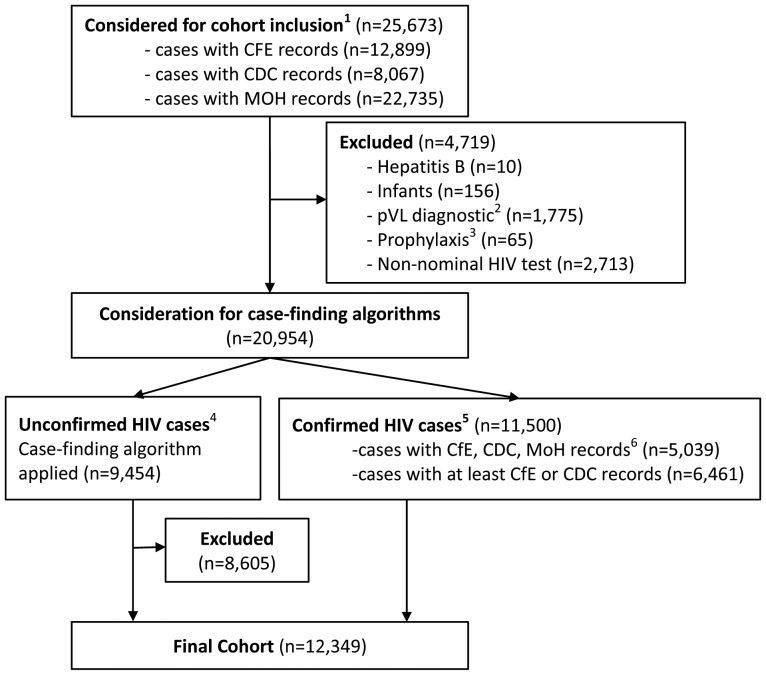
Flow diagram of the STOP HIV/AIDS cohort selection process. CDC: BC Centre for Disease Control; MoH: BC Ministry of Health Datasets (including Discharge Abstract Database, Medical Services Plan and PharmaNet databases); CfE: BC Centre for Excellence in HIV/AIDS. **†** identified with at least one record of the following: HIV positive test (CDC), HIV-related hospitalization or physician claim (MoH), pVL test, CD4 test, AIDS-defining illness or HAART dispensation (CfE). * Cases with one or more undetectable pVL tests, with no other HIV-related records. ** Identified with at least one HIV-related MoH record but no other HIV-related records in the CDC or CfE databases; *** Identified with confirmed positive HIV test, a pVL test with detectable viral load or antiretroviral dispensation.

The cohort selection process is illustrated in Figure 1. The initial cohort considered for inclusion totaled 25,673 individuals, identified in at least one of the 6 datasets outlined in Table 1. We excluded 10 (0.04%) individuals receiving treatment for Hepatitis B, 156 (0.6%) infants receiving HIV prophylaxis, with no HIV-related records after 18 months of age, 1,775 (6.9%) individuals with pVL tests evidently used as a diagnostic tool, 65 (0.3%) receiving pre- or post-exposure prophylaxis and 2,713 (10.6%) individuals with non-nominal positive HIV tests. The remaining cases consisted of 11,500 (50.1%) confirmed cases, of which 5,039 were designated as ‘gold standard’ cases, and 9,454 (41.2%) unconfirmed cases.

Results of the case-finding algorithms were presented in Table 2. Among unconfirmed cases, algorithm 2 identified 849 cases (9.0%) as HIV-positive, while algorithm 3, identified 1,665 (17.6%) cases. The sensitivity of these algorithms, determined amongst gold-standard cases, was 88% and 90% for algorithms 2 and 3, respectively. A more stringent algorithm (algorithm 1) featured substantially reduced sensitivity (78.4%), while a more lenient algorithm (algorithm 4) included all cases. These latter options were excluded from further consideration.

**Table 2:** Comparison of case-finding algorithms.

Algorithm Description	Identified cases among Unconfirmed HIV cases*	Algorithm sensitivity among gold standard HIV cases**
	N = 9,454	N = 5,039
Algorithm 1:	803 (8.5%)	3,951 (78.4%)
3 physician claims		
Algorithm 2:	849 (9.0%)	4,435 (88.0%)
3 physician claims OR 1 hospital admission		
Algorithm 3:	1,665 (17.6%)	4,537 (90.0%)
2 physician claims OR 1 hospital admission		
Algorithm 4:	9,454 (100.0%)	4,665 (92.6%)
1 physician claim OR 1 hospital admission		

CfE: BC Centre for Excellence in HIV/AIDS Drug Treatment Program or Laboratory Program Datasets. MoH: BC Ministry of Health datasets (including discharge abstract datasets (DAD - hospitalizations), PharmaNet (drug dispensation) and Master Services Plan (MSP - physician billing) datasets. CDC: BC Centre for Disease Control HIV testing dataset.

* Identified with at least one HIV-related MoH record but no other HIV-related records in the CDC or CfE databases;

** Identified with confirmed positive HIV test, a pVL test with detectable viral load or antiretroviral dispensation, and with records in each of the CfE, CDC and MoH databases.

We tested five hypotheses to guide the choice between algorithms 2 or 3 (Table 3). The cohort of individuals included in algorithm 3 but excluded in algorithm 2 [(algorithm 3: n = 1665)−(algorithm 2: n = 849) = 816] were more likely to be female (p<0.01), had lower mortality rates (p<0.01) and lower rates of health service utilization (9,745 days of medications dispensed/100PY versus 10,266/100PY (p<0.01); 602 physician billings/100PY versus 2,056/100PY (p<0.01); 29 days in hospital/100PY versus 98/100PY (p<0.01)) in comparison to the gold standard cohort. The cohort of individuals excluded in both algorithms 2 and 3 were similar to those included in algorithm 3 but excluded in algorithm 2, and statistically significantly different from gold standard cases in each of the above criteria.

**Table 3:** Comparison of demographics and health service utilization across categories of cases considered for inclusion into the STOP HIV/AIDS cohort.

	Excluded in algorithms 2, 3	P-value*	Included in algorithm 3, Excluded in algorithm 2?	P-value*	Included in algorithm 2	P-value*	Gold standard cohort**
N	7,889		816		849		5,039
*Demographics*							
Age [Median (IQR)]	41 (27, 59)	<0.01	42 (29, 59)	<0.01	44 (35, 55)	0.95	44 (38, 51)
Male gender [N (%)]	3,838 (49.2)	<0.01	454 (55.64)	<0.01	569 (67)	<0.01	3,904 (77.5)
Mortality rate	1.74/100PY	<0.01	2.18/100PY	<0.01	3.2/100PY	0.80	3.14/100PY
*Health Service Utilization*							
PharmaNet: days/100PY meds dispensed	8,514 (1,476, 29,338)	<0.01	9,745 (1,608, 31,718)	<0.01	16,335 (3,529, 44,123)	<0.01	10,266 (3,118, 25,268)
MSP: N/100PY of physician billings	548 (231, 1,234)	<0.01	602 (225, 1,460)	<0.01	702 (230, 2,013)	<0.01	2,056 (1,179, 3,341)
DAD: days/100PY in hospital	24 (10, 109)	<0.01	29 (10, 139)	<0.01	88 (20, 455)	0.89	98 (23, 335)

MSP: Medical Services Plan; CDC: BC Centre for Disease Control; MoH: BC Ministry of Health Datasets (including DAD, MSP); IQR: Interquartile range.

* Compared to the confirmed HIV cases with records in CfE, CDC, MoH databases.

** Identified with confirmed positive HIV test, a pVL test with detectable viral load or antiretroviral dispensation, and with records in each of the CfE, CDC and MoH databases.

? Included in algorithm 3 (N = 1665) Excluded in algorithm 2 (N = 849); 1665−849 = 816.

**Figure 2:**
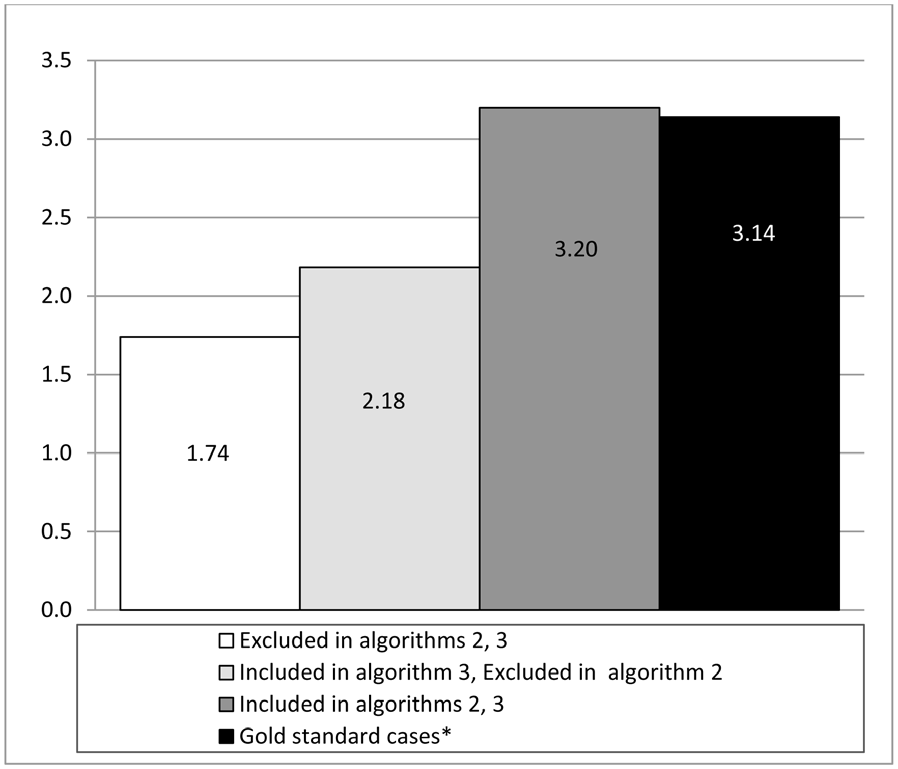
Mortality rates across categories of cases considered for inclusion into the STOP HIV/AIDS cohort. MoH: BC Ministry of Health; CfE: BC Centre for Excellence in HIV/AIDS; CDC: BC Centre for Disease Control. * Identified with confirmed positive HIV test, a pVL test with detectable viral load or antiretroviral dispensation.

While cases determined to be included in algorithm 2 (N = 849) were statistically significantly different from those excluded on each criterion on most dimensions (results not presented), these cases were more likely to be female (33% versus 22.5%; p<0.01) and received less outpatient care (702/100PY vs. 2056/100PY; p<0.01), but had higher levels of drug dispensation when compared to the gold standard cohort (16,335 days/100PY vs. 10,266/100PY; p<0.01) and rates of inpatient care that were not statistically significantly different from the gold standard cohort (88 days/100PY vs. 98/100PY; p = 0.89). Further, the mortality rate of these individuals was higher than that of the gold standard cohort (3.2/100PY vs. 3.14/100PY; p = 0.80), though the difference was not statistically significant (Figure 2). As a result, we selected algorithm 2 to define our cohort of HIV-positive individuals, thus adding 849 cases previously classified as unconfirmed, to 11,500 confirmed cases, for an overall sample size of 12,349 individuals with HIV/AIDS in British Columbia between January 1st, 1995 and March 31st, 2010.

## Discussion

We defined a cohort of individuals with HIV/AIDS in British Columbia from a systematic linkage of multiple population-level, province-wide health administrative datasets. Importantly, this cohort includes 3,576 (29%) individuals who had never accessed HAART, thus representing a critical target population requiring further study to inform efforts regarding engagement into HIV care. This new knowledge provides a rare opportunity to observe health care utilization patterns of HIV-positive individuals not engaged in regular HIV care, and provide a more complete basis for public health surveillance and monitoring.

Of note, the cohort represents a prevalence of 12,349 for the study period (1996–2010); at the end of March 2010, 9,597 individuals with HIV/AIDS remained in the study cohort, after excluding decedents using linked vital statistics data. In contrast, PHAC point prevalence estimated 11,040 individuals living with HIV/AIDS in BC in 2008, and 11,700 in 2011 [1], [2]; Despite substantial and increasing efforts to seek, test, treat and retain individuals with HIV/AIDS in BC, PHAC figures suggest 13–18% of these prevalent cases remain outside of the reach of the healthcare system and may have unknown HIV status. This information highlights the need to improve HIV testing strategies, and subsequently improve the ‘cascade of HIV care’ [10] in BC.

While the case-finding algorithms provided a structured means to select individuals for cohort inclusion, selecting the most relevant algorithm remains a subjective task. In the absence of a nested sub-group of cases and non-cases identified via chart review, the definition and testing of a priori hypotheses provide a means to confirm the results of the case-finding algorithms. In this application, the conclusiveness of these tests underlines their practical utility in applied settings. Further, individuals identified only through the administrative data may include some individuals diagnosed with HIV in BC but not linkable. This is a strength of this cohort in relation to others which have linked surveillance to treatment data only, and overcomes some of the known limitations due to incomplete identifiers in HIV surveillance data.

Over 90% of unconfirmed cases identified only in the MoH administrative databases were excluded using the selected case-finding algorithm, indicating a high frequency of coding errors related to HIV, particularly in the Medical Services Plan dataset. Further, we found no distinct trend in erroneous HIV coding, indicating a persistent, rather than deteriorating level of misclassification. Diagnostic coding errors are commonly reported in administrative physician billing records databases [17]–[20]; defining disease-based cohort based on such records requires careful consideration of inclusion/exclusion criteria and secondary validation.

The hypothesis tests provided strong evidence confirming the use of the chosen case-finding algorithms [14], which we found to be generalizable to a BC setting. These tests also provided an indication of the extent to which health service utilization would be underestimated using other means of cohort definition in this application. Individuals considered for cohort inclusion but excluded in algorithms 2 and 3 consumed, on average, 1,780/100PY fewer days of medication, spent 74/100PY fewer days in hospital, and had 1,508/100PY fewer physician billing records compared to gold standard cases. In contrast, those included in our analysis using the case-finding algorithms (algorithm 2) had higher rates of drug dispensation and mortality, and similar rates of hospitalization. These disparities serve to underscore the importance of the use of case-finding algorithms and subsequent confirmation or validation in defining cohorts from administrative databases for health services research, particularly in diseases likely to result in high levels of health resource use such as HIV/AIDS [25], [26].

There were several limitations in the analyses presented. The inability to confirm, with certainty, the diagnosis of all selected cases was inherent and is indeed the premise of the manuscript. Nonetheless, we've outlined a pragmatic approach for cohort selection using health administrative datasets. Misclassification of unconfirmed HIV cases remains possible, and HIV-positive individuals with no HIV-related contact with the BC health system could not be captured. Our inability to link non-nominal HIV tests likely resulted in a smaller sample than otherwise possible, and may have resulted in misclassification of cases not linked to HIV care as some unlinked cases may have been captured among the confirmed and unconfirmed cases. A separate analysis by CDC database managers found unlinked (non-nominal) cases (N = 2,094) were of younger age, more likely to be men who have sex with men, more likely to have resided in the Vancouver Coastal Health Authority region, and were diagnosed early in the study period (results not presented). Also, in-and-out migration was not observed, and therefore was not accounted for in our analysis. A recent BC study indicated a high level of within-province migration - nearly 50% of all individuals in treatment had migrated between local health areas during a median 3.9 years of follow-up [27] – however in- and out-of-province migration could not be measured. We expect in-migration, and transient individuals may inflate the number of individuals included in the cohort, while out-migration, resulting in an unobserved censorship, may result in under-estimates of health resource utilization in subsequent analyses. Furthermore, periods of incarceration in provincial and federal corrections facilities were not captured within the available datasets, which may result in underestimated rates of health service utilization among included cases. Further study and efforts to establish additional data linkage are underway to address these limitations.

In conclusion, as electronic medical records become more commonplace, the availability of large administrative and clinical databases for programmatic monitoring and evaluation, as well as for research purposes is likely to expand. In this analysis, we have demonstrated the ability to identify HIV-infected subjects in the HAART era using an existing algorithm, and validated this algorithm with a series of a priori hypothesis tests.
